# Audiological findings in pacients treated with radiotherapy for head and neck tumors

**DOI:** 10.1590/S1808-86942010000400019

**Published:** 2015-10-19

**Authors:** Ana Helena Bannwart Dell'Aringa, Myrian de Lima Isaac, Gustavo Viani Arruda, Alfredo Rafael Dell'Aringa, Maria Carolina B.N. Esteves

**Affiliations:** a Audiologist; Speech and Hearing Therapist - Otolaryngology Program - Medical School of Marília; b PhD in Otorhinolaryngology, Professor of Otorhinolaryngology - Medical School of the University of São Paulo in Ribeirão Preto, FMUSP-RP; c Expert in radiology, Professor of Oncology and Radiotherapy of the Medical School in Marilia; d PhD in Otorhinolaryngology - Head of the Otolaryngology Program - Medical School of Mariilia; e Expert in Otorhinolaryngology - volunteer physician - Department of Otorhinolaryngology - Medical School of Marilia

**Keywords:** head and neck neoplasms, loss, radiotherapy

## Abstract

Radiotherapy has been widely used given its increase in the successful outcomes and cure of some cancers.

**Aim:**

To evaluate the functionality of the auditory system in patients who underwent radiotherapy treatment for head and neck tumors.

**Materials and Methods:**

From May 2007 to May 2008, otorhinolaryngological and audiological evaluation (Pure Tone Audiometry (air and bone conduction), Speech Audiometry, Tympanometry, Acoustic Reflex testing and Distortion Product Otoacoustic Emissions) were performed in 19 patients diagnosed with head and neck neoplasia and treated with radiotherapy. Prospective case series study.

**Results:**

10.5% left ears and 26.3% right ears had bilateral hearing loss soon after radiotherapy according to ASHA criteria.

**Conclusions:**

Radiotherapy treatment for head and neck cancer has ototoxic effects. Early programs of auditory rehabilitation should be offered to these patients.

## INTRODUCTION

Hearing loss is one of the main complications of head and neck cancer tumors^1^. Recently, adding chemotherapy (QT) with cisplatin to radiotherapy (RT) has enhanced the survival of patients with such neoplasia, thus becoming the standard treatment for locally advanced tumors. Nonetheless, all types of treatment, radiotherapy and chemotherapy with cisplatin, are known for their ototoxic effects.

Irreversible hearing loss, as a consequence of head and neck radiotherapy has been studied and the literature shows a great variability in the incidence of ototoxicity, varying between 18% and 50%.^2^, ^3^, ^4^, ^5^

Insofar as the ototoxic effects caused by radiation to the head and neck are concerned, the literature is full of controversies.

Smouha and Karmody reported external auditory meatus necrosis, osteoradionecrosis of the temporal bone, otitis media, conductive hearing loss, otalgia and tinnitus as main consequences.^6^

Bohne et al.^7^ reported that the degeneration of hair cells, both sensorial and support cells may happen up to two years after the end of radiotherapy. Other authors have reported a bilateral sensorineural deficiency in the high frequencies as a consequence to the radiation.^8^, ^9^, ^10^

Our study aimed at assessing the auditory system function in patients submitted to radiotherapy in the head and neck region because of the concern with its ototoxic effects.

## MATERIALS AND METHODS

Prospective study carried out at the Department of Otorhinolaryngology and Oncology/Radiotherapy, approved by the Ethics in Research Committee, under protocol # 165/06.

We had a total of 19 male patients diagnosed with head and neck neoplasia enrolled in this study.

Because of primary tumor characteristics and clinical staging, all the patients were treated with radiotherapy.

After confirming the diagnosis of neoplasia through a pathology exam and indication of radiotherapy as treatment, the patients were referred to the ENT ward for otolaryngological and audiological evaluation, made up in two stages: before and after radiotherapy. The following procedures were held: Clinical-ENT anamneses and evaluation, speech and hearing therapy interview, air conduction and bone tonal threshold audiometry, logoaudiometry, immittance evaluation, stapes reflex study and distortion product evoked otoacoustic emissions (DP-EOAE).

Previous chemo and/or radiotherapy treatment, whether concurrent or not was considered as an exclusion factor, as well as patients with rhinopharynx neoplasia.

We took off the sample two patients with this type of neoplasia because of the presence of unilateral pretreatment conductive hearing loss (neoplasia side) and normal post-treatment hearing (probably due to a reduction in tumor size and Eustachian tube decompression).

In the radiotherapy program, all the patients were staged by means of a complete physical exam, direct or indirect laryngoscopy, head and neck CT scan and chest x-ray before treatment.

The dose of radiotherapy prescribed varied according to the disease's primary site and primary tumor staging. Before starting the radiotherapy sessions, the patients were submitted to conventional simulation (x-Ray), in order to outline the radiotherapy field and make up of moldable thermoplastic masks used for immobilization in the supine position and to outline the treatment area.

Treatment limits (radiation field) vary according to the disease's primary site. Nonetheless, because of the advanced nature of the cases, all the patients had the upper border of the radiotherapy field on the skull base, which resulted in the inclusion of the cochlea in the radiotherapy field.

In order to calculate the radiotherapy treatment volume (RTV) we used the patient's side-to-side distance, measured during the process of simulation and multiplied by the resulting area of simulation in each patient.

### Statistical analysis

In order to analyze the differences between the tonal threshold mean values for the different frequencies before and after treatment, we did the t-student test.

In order to check and see which mean values (above or below) for age, volume and RT dose variables would have some association with the reduction of the tonal thresholds after treatment we employed the Fisher's Exact test; and according to the contingency table we calculated the Odds Ratio in order to establish the relationship between the likelihood of occurrence and non-occurrence of a post-treatment auditory change, considering the variables: age, volume and RT dose.

As a reduction of hearing acuity we considered the ASHA criteria which considers a 20dB increase in ototoxicity effect in one isolate frequency or of 10 dB in two or more successive frequencies.^11^

For all the statistical tests, we considered the value up to 5% for significance level (p < 0.05).

## RESULTS

The patients' characteristics, including gender, age, tumor anatomical location, distribution by clinical staging, radiotherapy dose by fraction, total dose of radiotherapy and radiotherapy treatment volume are described on Chart 1.

**Chart 1:** Distribution of the individual characteristics of each patient from Group 1 (n = 19).

	Gender	Age	Anatomical Location	Staging	Fractioned dose	Total dose	Treatment volume
1	M	66	Parotid	T4N0M0	2,0	60,0	768
2	M	64	Larynx	T4N0M0	1,8	70,2	1.261
3	M	73	Oropharynx	T4N3M0	1,8	70,2	2.828
4	M	62	Larynx	T3N0M0	2,0	72,0	1.562
5	M	66	Parotid	T1N1M0	2,0	60,0	1.411
6	M	59	Oropharynx	T2N3M0	1,8	72,0	2.608
7	M	52	Larynx	T2N0M0	2,0	66,0	1.664
8	M	45	Oropharynx	T3N0M0	2,0	60,0	992
9	M	52	Oropharynx	T4N0M0	1,8 −2,0	61,0	1.930
10	M	64	Oropharynx	T2N1M0	1,8	61,2	1875
11	M	66	Larynx	T4N2M0	2,0	72,0	2.044
12	M	77	Larynx	T2N0M0	1,8	66,6	1.822
13	M	74	Larynx	T4N3M0	1,8	72,0	1.980
14	M	58	Oropharynx	T2N0M0	2,0	60,0	1.605
15	M	78	Larynx	T4N0M0	2,0	64,0	1.060
16	M	58	Larynx	T4N0M0	1,8	60,0	1.597
17	M	76	Hypopharynx	T2N0M0	1,8 −2,0	72,0	2.400
18	M	82	Oropharynx	T2N1M0	2,0	62,0	1.905
19	M	37	Larynx	T2N0M0	1,8	66,6	1.822

As seen on Chart 1, all the patients in this group were males in the age range between 37 and 82 years, and the mean age was 63.6 years and median of 64 years. The total radiotherapy dose varied between 60.0 Gy and 72.0 Gy - mean dose of 65.6 Gy and median dose of 64 Gy, with daily dose variation between 1.8Gy −2,0 Gy. As to radiotherapy mean treatment volume we found 1,743.8 cm^3^, median of 1,664 cm^3^ and a variation of 768 cm^3^ to 2,828 cm^3^.

Among the 19 patients and 38 ears evaluated we noticed through the ENT evaluation that one ear had perforated tympanic membrane and 37 tympanic membranes were intact. During the radiotherapy sessions, only one ear showed otoscopic changes, and this membrane was intact before treatment and it was retracted after it. In the first audiologic evaluation this ear had sensorineural auditory loss and after treatment the loss was mixed.

As to the audiological evaluations performed before the radiotherapy treatment we noticed in our sample that only 4 ears had thresholds within the normal ranges (lower than 20 dB in all the frequencies) and most of the assessed patients already had some type of hearing disorder. One ear had moderate mixed hearing loss.

The remaining ears had sensorineural changes: moderate hearing loss (8 ears); mild hearing loss (5 ears), hearing loss starting at 2KHz (2 ears), starting at 3KHz (8 ears), starting at 4KHz (7 ears) and starting at 6KHz (3 ears).

By analyzing the data we can see that the mean values of the frequencies assessed between 250Hz and 8 KHz were changed when pre and post radiotherapy treatments were compared, as shown on Chart 2, which also shows data concerning standard deviation, confidence interval and p-value for each frequency bilaterally.

**Chart 2:** Distribution of the tonal auditory thresholds and the difference among them before and after treatment, confidence interval and p-value for each frequency in both ears (n=19).

	Frequency	Pretreatment mean value	Post-treatment mean value	Difference between the mean values	Confidence interval	p-value
	250 Hz	21,57	23,15	1,58	−6,12 2,96	0,47
	500 Hz	19,47	22,10	2,63	−7,47 2,21	0,26
	1 KHz	21,84	23,68	1,84	−6,40 2,71	0,40
RE	2 KHz	27,89	31,31	3,42	−8,44 1,59	0,16
	3 KHz	37,11	42,36	5,25	−12,00 1,48	0,18
	4 KHz	45,00	52,89	7,89	−15,31 −0,47	0,38
	6 KHz	62,10	55,00	−7,10	−0,27 14,48	0,58
	8 KHz	62,89	55,79	−7,10	0,13 14,07	0,04
	250 Hz	21,84	21,57	−0,27	−2,57 3,10	0,84
	500 Hz	17,63	17,89	0,26	−2,86 2,33	0,83
	1 KHz	18,68	20,78	2,10	−4,27 0,06	0,05
LE	2 KHz	26,05	27,63	1,58	−3,85 0,70	0,16
	3 KHz	36,57	34,47	−2,10	−0,82 5,03	0,14
	4 KHz	45,26	43,68	−1,58	−1,32 4,48	0,26
	6 KHz	53,15	51,31	−1,84	−2,02 5,71	0,33
	8 KHz	55,00	62,89	7,89	−14,18 −1,60	0,01

According to ASHA criteria, 10.5% left ears and 26.3% right ears had reduction in their tonal auditory thresholds immediately after the end of the radiotherapy. Post-treatment audiological evaluations were carried out within 15 days after the last radiotherapy session.

Age, radiotherapy treatment value and total radiation dose according to Fisher's exact test analyses did not show relationship with a reduction on the post-treatment tonal auditory thresholds when ASHA criteria were considered (Chart 3).

**Chart 3:** Data from the relationship between the likelihood of occurrence and non-occurrence of post-radiotherapy hearing loss, taking into account the variables: age, total dose of radiation and volume of radiotherapy treatment volume with ASHA criteria.

			Age		Total	p-value
			≤ 64 years	> 64 years		
	Hearing loss	Yes	8	7	15	0,667
	ASHA	No	2	2	4	
Total			10	9	19	
			RT total dose		Total	p-value
			≤ 64 Gy	> 64 Gy		
	Hearing loss	Yes	1	2	3	0,41
	ASHA	No	8	16	24	
Total			9	18	27	
			RT Treatment volume		Total	p-value
			≤ 1800 cm	> 1800 cm		
	Hearing loss	Yes	1	3	4	0,333
	ASHA	No	8	7	15	
Total			9	10	19	

## DISCUSSION

Radiotherapy is one of the effective treatment modalities for head and neck tumors.

For initial T1 and T2 lesions the results for RT alone are comparable to those obtained from surgical treatment. For more advanced lesions, such as some head and neck anatomical sites, RT associated with QT has been preferred because of the possibility of preserving the organ. Radiotherapy complications happen in specific sites, in other words, they depend on the area (radiotherapy field) where the radiation is being deployed.^12^

Especially for advanced disease, because of the presence of large tumors, there is the need to irradiate the primary site of the disease and those areas suspected of microscopic disease, having potential side effects as consequences, especially on the healthy tissue near the tumor site. As an example of this we have ototoxicity in the cases of head and neck RT, especially when the cochlea is inside the radiation field.

Although we do not do 3-D image shaped RT, we were able to estimate the RTV for each patient using the technical parameters for simulation and treatment. In a statistical analysis we compared the group of patients who received RTV in greater or lower than 1,800 cm^3^, and no significant auditory changes were seen among them. Therefore, for the sample investigated, the radiotherapy treatment volume was not considered an important risk factor for ototoxicity (Chart 3).

As to the radiation dose factor, Che et al. reported a relationship between ototoxicity and the punctual dose of radiation received by the cochlea. When using the 3-D shaped RT, there was a significant increase in the risk of acquiring hearing loss in patients who received radiation doses starting from 48 Gy.^13^

According to results from our statistical analyses, we did not observe significant association regarding tonal auditory thresholds after treatment for patients who received radiation doses higher than 64 Gy. It is worth stressing that this is the total radiation dose administered to the treatment field and not the dose received by the cochlea (Chart 3).

The increase in the age of the patients submitted to treatment has also been shown as a factor involved in the increase of risk for hearing reduction. Some studies associate age increase with the increase in ototoxicity.^14^^,^^15^

In our sample, all the patients assessed had ages ≥ 40 years, and the mean age of 64 years. In a statistical analysis we also did not notice statistically significant differences between the groups of patients with ages above and below 64 years (Chart 3).

As to changes in tonal auditory thresholds immediately after radiotherapy, we find in our sample an increase in tonal thresholds for the frequencies of 4 KHz in the right ear and 8 KHz in the left ear.

All the patients in our sample had hearing disorders; 36.8% of them had significant changes according to ASHA ototoxicity criteria and 63.2% had threshold increases which did not fit these criteria.

In a prospective study, Ho et al. assessed 526 ears from patients with nasopharynx cancer treated by radiotherapy alone.^16^ With a follow up of 4.5 years they observed that the hearing changes started immediately after the end of the radiotherapy. After two years, 40% of the patients partially recovered from their hearing change, while the remaining of the patients had a worsening as the years passed.

We stress that nasopharyngeal neoplasias may cause conductive hearing loss because of Eustachian tube compression.

In regards of the auditory complaints presented by the patients, some studies report tinnitus, while others report that tinnitus is usually transitional, disappearing within hours or weeks after the treatment and it happens to 2% to 36% of the patients.^17^, ^18^, ^19^. In our sample, only one patient complained of tinnitus during and after radiotherapy. Three patients already complained of tinnitus before the treatment and did not show changes during treatment. One patient complained of post-treatment otalgia.

## CONCLUSION

Patients with head and neck cancer submitted to conventional radiotherapy have a high incidence of tonal hearing threshold increases at the end of treatment.

Our data stress the importance of doing a pre and post treatment audiologic evaluation in all the patients submitted to conventional radiotherapy to treat head and neck tumors.

Thus, we stress the importance of these instructions on the consequences of radiotherapy, so that the patients can be included early on in hearing rehabilitation programs.

## FINAL REMARKS

All the patients with hearing reduction and who reported hearing problems were instructed regarding the use of hearing aids.
